# Functional characterization of the vertebrate primary ureter: Structure and ion transport mechanisms of the pronephric duct in axolotl larvae (Amphibia)

**DOI:** 10.1186/1471-213X-10-56

**Published:** 2010-05-27

**Authors:** Birgitte M Haugan, Kenneth A Halberg, Åse Jespersen, Lea R Prehn, Nadja Møbjerg

**Affiliations:** 1 Department of Biology, University of Copenhagen, Universitetsparken, DK-2100 Copenhagen, Denmark

## Abstract

**Background:**

Three kidney systems appear during vertebrate development: the pronephroi, mesonephroi and metanephroi. The pronephric duct is the first or primary ureter of these kidney systems. Its role as a key player in the induction of nephrogenic mesenchyme is well established. Here we investigate whether the duct is involved in urine modification using larvae of the freshwater amphibian *Ambystoma mexicanum *(axolotl) as model.

**Results:**

We investigated structural as well as physiological properties of the pronephric duct. The key elements of our methodology were: using histology, light and transmission electron microscopy as well as confocal laser scanning microscopy on fixed tissue and applying the microperfusion technique on isolated pronephric ducts in combination with single cell microelectrode impalements. Our data show that the fully differentiated pronephric duct is composed of a single layered epithelium consisting of one cell type comparable to the principal cell of the renal collecting duct system. The cells are characterized by a prominent basolateral labyrinth and a relatively smooth apical surface with one central cilium. Cellular impalements demonstrate the presence of apical Na^+ ^and K^+ ^conductances, as well as a large K^+ ^conductance in the basolateral cell membrane. Immunolabeling experiments indicate heavy expression of Na^+^/K^+^-ATPase in the basolateral labyrinth.

**Conclusions:**

We propose that the pronephric duct is important for the subsequent modification of urine produced by the pronephros. Our results indicate that it reabsorbs sodium and secretes potassium via channels present in the apical cell membrane with the driving force for ion movement provided by the Na^+^/K^+ ^pump. This is to our knowledge the first characterization of the pronephric duct, the precursor of the collecting duct system, which provides a model of cell structure and basic mechanisms for ion transport. Such information may be important in understanding the evolution of vertebrate kidney systems and human diseases associated with congenital malformations.

## Background

During the development from embryo to adult life vertebrates use a succession of kidney forms to maintain extracellular fluid homeostasis and simultaneously rid the body of nitrogenous wastes [1,2]. Three spatially and temporally different kidney generations form from the intermediate mesoderm in an anterior to posterior direction i.e. the pronephroi, mesonephroi and metanephroi [3]. The functional unit in these paired kidneys is the nephron, and it is composed of a filtration unit and a renal tubule. Urine is produced by the filtration of blood in the filtration unit, followed by the selective reabsorption and secretion of ions, organic molecules and water across highly specialized epithelia of the renal tubule [4]. Nephrons open into a ureter, and in the meso- and metanephros they do so via a collecting duct system [5,6]. This system is the site for the important final adjustment of the urine. Vertebrate kidneys may produce urine, which is either hypoosmotic (diluted), isoosmotic or hyperosmotic (concentrated) relative to the body fluids [7-10]. This ability is a function of *i*) the evolutionary state of the nephrons and *ii*) the regulation of filtration and of the transport of inorganic ions, organic molecules and water across the renal epithelia.

The first kidneys to form - the embryonic pronephric kidneys - are the functional kidneys of fish and amphibian larvae [11-16]. These are very simple kidneys composed of a single nephron. A characteristic of vertebrate kidney organogenesis is the development of a pronephric duct in association with each pronephros [3]. These ducts are the first or primary ureters of vertebrate kidney systems. They form the collecting duct system of the meso- and metanephroi, and they, and their derivates, are the key players in the induction of the nephrogenic mesenchyme, which forms these latter kidney generations. Few functional studies exist on the pronephros and functional studies of the duct are virtually lacking. Molecular studies have been directed at mapping genes expressed in different segments of the pronephric nephron, and several recent reviews have highlighted the potential use of this embryonic kidney in drug and human kidney disease assessment [17-23]. To date, there has been little focus on the role of the pronephric duct in urine modification and it remains to be shown whether transepithelial transport processes are present in this structure. Ultrastructural investigations have shown duct cells with the characteristics of an epithelium involved in active transport e.g. many mitochondria and surface expansions of the basolateral cell membranes [14,24]. In addition, gene expression assays have indicated high expression of transporters known to be involved in ion transport, such as the Na^+^/K^+^-ATPase and the ROMK channel [17,18,25-27]. Collectively, these data suggest that the pronephric duct may play an important role in regulation of extracellular fluid homeostasis. Therefore we ask the question: Is the duct involved in urine modification?

In amphibians the pronephros is a large organ, which is functional for a considerable time, before it degenerates. We investigate structural and functional characteristics of the pronephric duct in the freshwater amphibian *Ambystoma mexicanum *(axolotl). Members of the *Ambystoma *genus have been used as models for the study of pronephric structure, function, development and evolution for more than a century [24,28,29] and the formation and caudal migration of the pronephric duct in the axolotl has been thoroughly investigated [30-32]. Numerous functional studies exist on the mesonephric collecting duct system of both urodele and anuran amphibians, which provide detailed information on the transport characteristics of these segments [Reviewed in [13]]. Our histological examinations and dissections of axolotl larvae indicate that the pronephros is functional from the time of hatching to larval stage 54. We investigate duct morphology and cellular transport mechanisms present in larvae with functional pronephroi, and show that the primary ureter is important for urine dilution in the axolotl. The single cell type found in the ureter shows the characteristics of the vertebrate collecting duct system principal cell and our data indicate that it reabsorbs Na^+ ^and secretes K^+^.

## Results and Discussion

### Identification of functional pronephroi and pronephric ducts

**Figure 1:**
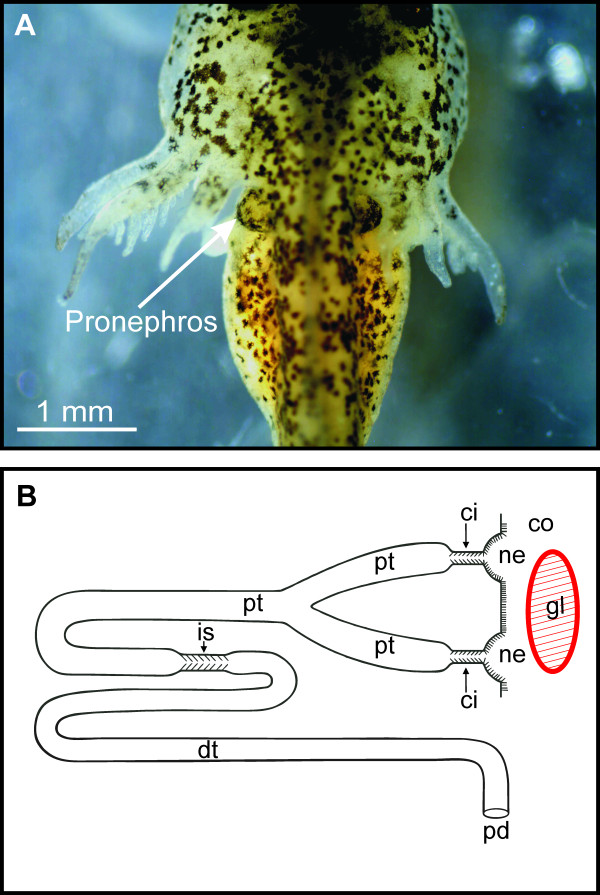
**Axolotl larva stage 45 and 52**. **A**. Dorsal view of recently hatched axolotl larva (stage 45; forelimb present as limb buds). The pronephroi can be seen as two small bulges behind the gills. **B**. Schematic representation of the pronephric nephron in the stage 52 larva as revealed from light- and transmission electron microscopy on serial section of plastic embedded tissue. The pronephros consists of an external glomus (gl) and a single renal tubule, which opens into the coelom (co) via two ciliated nephrostomes (ne). In this late larval stage the tubule is divided into two ciliated tubules (ci), two proximal tubule branches (pt), a common proximal tubule, a ciliated intermediate segment (is) and a distal tubule (dt). The distal tubule continues as the pronephric duct (pd), which leaves the confines of the pronephros and empties into the cloaca.

**Figure 2:**
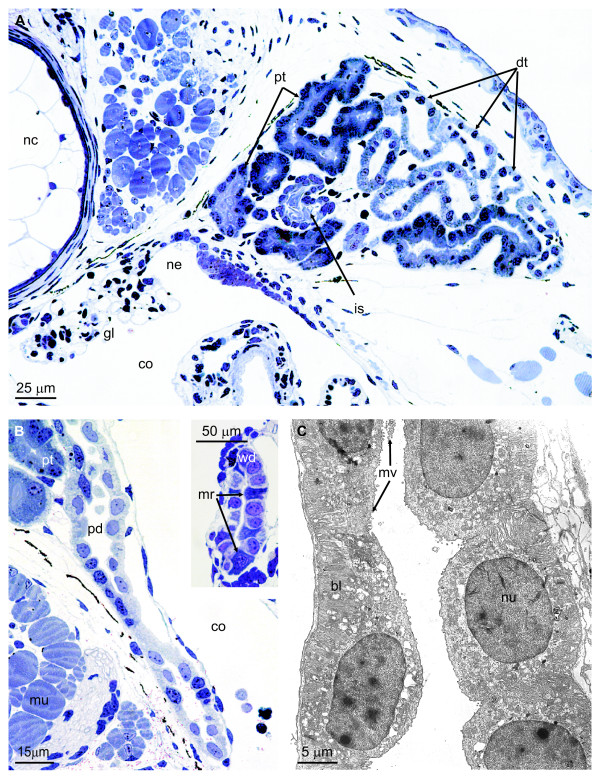
**Histology of pronephros and pronephric duct**. **A**. Cross section of a stage 54 larva (forelimb completely developed) revealing the filtration unit and the convoluted pronephric tubule. Araldite section, 1.5 μm, stained with toluidine blue. Blood is filtered in the external glomus (gl) and the filtrate enters the coelom (co) before it is taken up into the renal tubule via ciliated nephrostomes (ne). In this late larval stage the tubule is characterized by possessing a ciliated intermediate segment (is). nc, notochord; dt, distal tubule; pt, proximal tubule. **B**. Longitudinal section of pronephric duct (stage 52 larva). Araldite section, 2 μm, stained with toluidine blue. The pronephric duct (pd) leaves the confines of the pronephros. co, coelom; mu, muscle; pt, proximal tubule. INSERT: The Wolffian duct (wd) at the level of the caudal part of the mesonephros in a stage 54 larva. The duct epithelium consists of two cell types: principal cells and intercalated, mitochondria-rich cells (mr). **C**. Transmission electron microscopy of pronephric duct shown in figure 2B. The duct is composed of a single cell type characterized by a relative smooth apical surface with few microvilli (mv) and a well developed basal labyrinth (bl) formed by the highly invaginated basal and to some extent lateral cell membranes. nu, nucleus.

We determined the interval in which the axolotl pronephros and pronephric duct is functional by investigating kidney structure in freshly dissected larvae and in larvae prepared for histology (Figure 1 and 2). The pronephroi of axolotl larvae are paired organs located on each side of the dorsal aorta in the most anterior part of the body cavity. They are visible from the outside on the dorsal side of the larva as two small bulges behind the gills (Figure 1A). Each of the two kidneys are composed of a filtrating unit - a glomus originating from the dorsal aorta, and a single convoluted renal tubule opening into the coelom via two ciliated nephrostomes (Figure 1B). The pronephros is fully functional when the axolotl larva hatches from the egg at stage 44. This was confirmed by the appearance of blood cells in the capillaries of the glomus, which from embryonic stage 36 had a fully developed endothelium and a visceral layer with podocytes. From stage 44 the cilia of the nephrostomes points toward the lumen of the renal tubule, indicating a passage of fluid from the coelom. At this stage the kidney consists of an external glomus and a renal tubule with the following morphologically determined segments: two nephrostomes, each connected to a branch of proximal tubule, a common proximal tubule and a distal tubule. The pronephric duct runs caudally as an extension of the distal tubule opening into the cloaca. From the time of hatching the kidney was observed to increase in overall size due to further segmentation of the renal tubule i.e. the development of a ciliated intermediary segment; present from stage 52 (Figure 1B and 2A). The fully segmented pronephric tubule consists of the following morphological defined segments: two nephrostomial tubules, two proximal tubule branches, a common proximal tubule, a ciliated intermediary segment and a distal tubule (Figure 2A). At stage 52 the mesonephros was clearly visible and functional as judged by glomerular maturation and presence of blood cells in the mesonephric glomerular capillaries. Hence, comparable to the situation found in anuran amphibians [14,33] the two kidney generations functionally overlap in axolotl larvae. At stage 54 the pronephros reaches its maximal size. Gonadal primordia were observed medioventral to the mesonephros (not shown). These were undifferentiated and sex determination was not possible. During the transition from stage 54 to latter stages, characterized by fully developed hind limbs, overall pronephric tubule and glomus volume decreased, marking pronephric degeneration. Pronephric structure in stage 52-54 larvae of *Ambystoma mexicanum *resembled the description by Christensen (1964) of the functional pronephros in *A. punctatum *[24].

### Pronephric duct structure

Structural examination of the pronephric duct in larvae with functional pronephroi revealed that the duct consists of a single cell type (Figure 2B and 2C). Thus, the heterocellularity with intercalated mitochondria-rich cells interposed between principal cells, characteristic of the collecting duct system of latter kidney generations, is not seen at this point (Figure 2B and 2C). This is comparable to the situation in the green toad, *Bufo viridis *- a terrestrial anuran amphibian [14]. The pronephric duct cells in *A. mexicanum *are approximately 20 μm high in early larval stages, but decrease in height to 10-15 μm in stage 52 and 54 larvae (Figure 2C). They have a relatively smooth apical surface. A single central cilium is present (not shown) in addition to small and sparse microvilli, which are more numerous at the point of the apical cell junctions. The nucleus is regularly shaped, centrally placed and contains a nucleolus and patches of heterochromatin. There is a conspicuous basal labyrinth, and lateral infoldings are seen as well (Figure 2C). The cytoplasm contains many mitochondria in addition to a Golgi complex and endoplasmatic reticulum. The morphology of the duct changes at the level of the mesonephros in late larval stages 52-54, revealing the presence of intercalated, mitochondria-rich cells (Figure 2B, insert).

### Ion transport mechanisms in the pronephric duct of larvae stage 46-54

**Figure 3:**
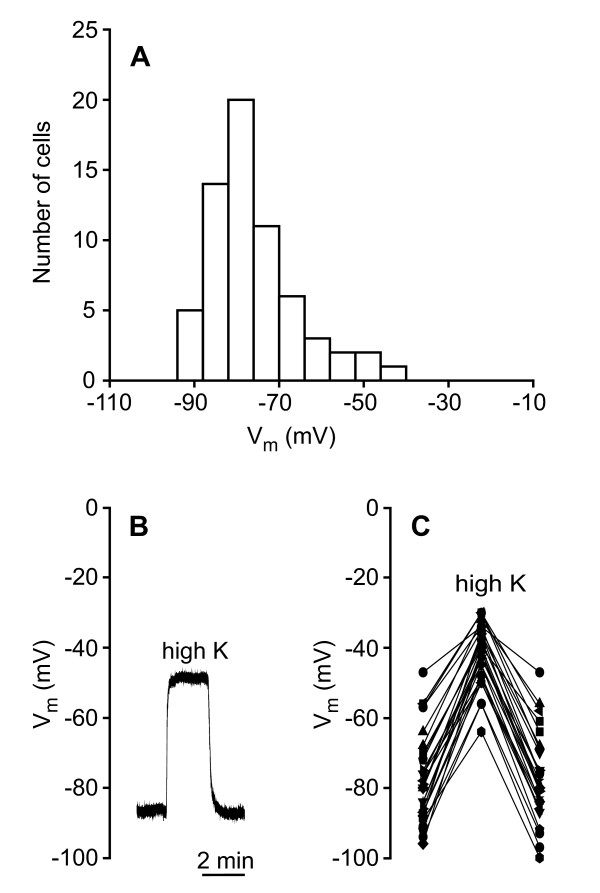
**Voltage recordings from single cells in isolated and perfused ducts**. **A**. Frequency distribution of the membrane potential (V_m_) in 64 cells from pronephric ducts dissected from axolotl larvae in the stage 46-54. **B**. Original voltage trace from single cell. Effect of raising bath [K^+^] from 3 to 20 mmol/l. V_m _depolarized indicating the presence of a basolateral K^+ ^conductance. **C**. Summary data illustrating the effect on V_m _of the bath K^+ ^concentration step (n = 29).

**Figure 4:**
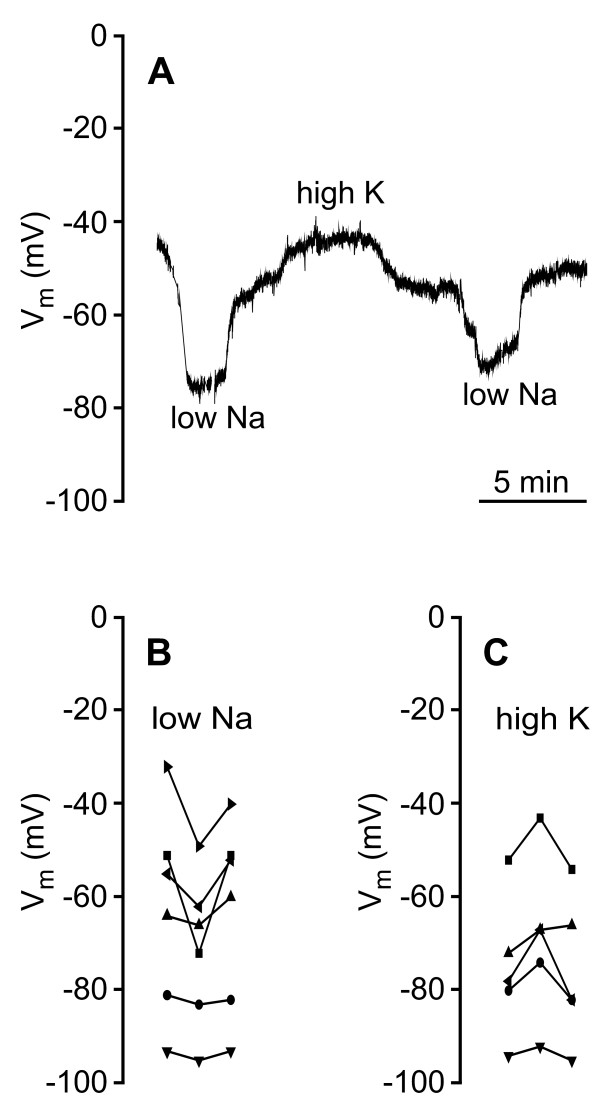
**Electrophysiological response to luminal fluid exchange**. **A**. Original voltage trace from single cell. Effect of changing luminal [Na^+^] from 102 to 7 mmol/l and luminal [K^+^] from 3 to 20 mmol/l. V_m _hyperpolarized upon the decrease in luminal [Na^+^] and depolarized upon an increase in luminal [K^+^]. This indicates that the luminal (apical) cell membrane possesses Na^+ ^as well as K^+ ^conductances. **B**. Summary data illustrating the effect on V_m _of concentration steps in luminal Na^+ ^(n = 6) and K^+ ^(n = 5).

**Figure 5:**
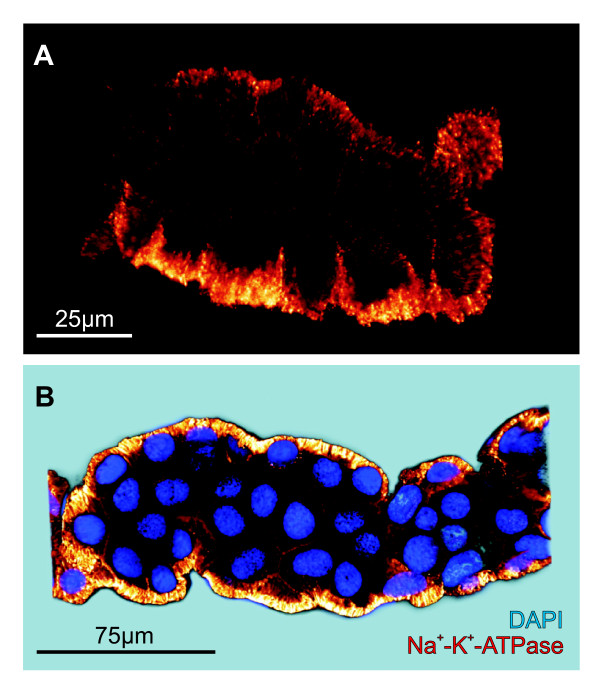
**Na^+^-K^+^-ATPase expression**. **A**. The Na^+^-K^+^-ATPase is highly expressed in the pronephric ducts and entirely localized to the lateral and highly invaginated basolateral cell membranes. **B**. Na^+^-K^+^-ATPase expression in duct counterstained with DAPI. Images are three-dimensional reconstructions of the original CLSM z-series, showing a median longitudinal section of the pronephric duct.

We examined if the pronephric duct participates in final urine modification with the aid of glass microelectrodes and ion substitution experiments in isolated and perfused ducts dissected from 22 larvae. Figure 3A is a frequency distribution of the membrane potential (V_m_) of 64 impaled cells. The data show a broad distribution with an average V_m _between -75 and -80 mV. Transport characteristics of the duct did not seem to differ between early and late larval stages. As shown in figure 3B and 3C, raising the K^+ ^concentration in the basal solution from 3 to 20 mmol/l resulted in a large, reversible depolarization of V_m_, revealing the presence of a large basolateral K^+ ^conductance. This indicates the presence of K^+ ^channels in the basolateral cell membrane. We examined whether the duct has luminal electrogenic transporters or channels in experiments with luminal K^+ ^and Na^+ ^steps. Figure 4 shows the result of these substitution experiments. V_m _hyperpolarized upon a decrease in luminal Na^+ ^concentration from 102 to 7 mmol/l and depolarized upon an increase in luminal K^+ ^concentration from 3 to 20 mmol/l. This indicates that the luminal (apical) cell membrane possesses Na^+ ^as well as K^+ ^channels. In order to identify an ion pump, which can provide the driving force for luminal uptake of Na^+ ^as well as K^+ ^secretion, we isolated pronephric ducts and performed immunolabeling with an antibody directed against the Na^+^-K^+^-ATPase α-subunit. As shown in figure 5, the Na^+^-K^+^-ATPase is highly expressed in the pronephric ducts from these larvae, and is entirely localized to the lateral and highly invaginated basolateral cell membranes.

Electrophysiological studies performed on the mesonephric collecting duct system of amphibians, have indicated that principal cells in aquatic urodeles, have a large apical Na^+ ^conductance and no, or very small, K^+ ^conductance. However, in terrestrial anurans, K^+ ^secretion through apical K^+ ^channels seems a major task of the principal cells [34-39]. In the current study we provide evidence for a principal cell, which has the characteristics of the mammalian collecting duct principal cell, i.e. with luminal Na^+ ^as well as K^+ ^conductances.

## Conclusions

**Figure 6:**
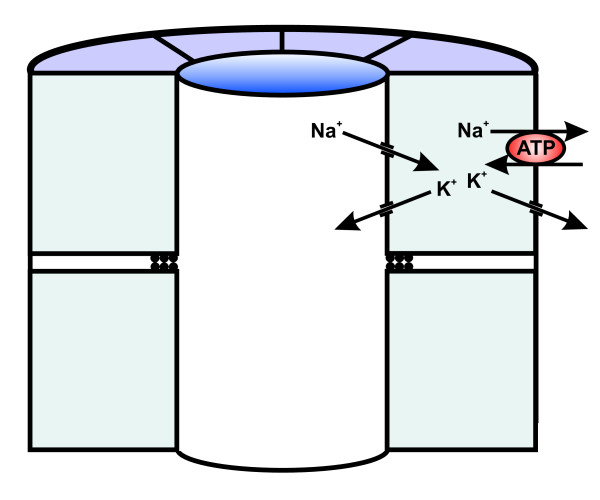
**Suggested model for ion transport mechanisms in the cells of the vertebrate primary ureter**. A Na^+^-K^+^-ATPase in the basolateral cell membrane pumps Na^+ ^out of the cell and thereby provides the driving force for apical uptake of Na^+ ^through channels. K^+ ^is secreted across the apical cell membrane through channels and recycled for the pump across the basolateral cell membrane.

We show that the pronephric duct, which is the first or primary ureter in all vertebrates, participates in urine adjustment in the axolotl. The cells constituting the duct are on the ultrastructural as well as cell physiological level comparable to principal cells found in the first segments of the mammalian collecting duct system [5,6,14,40-42]. Notably, the pronephric duct lacks intercalated, mitochondria-rich cells. We propose that the duct is important for urine dilution through NaCl reabsorbtion, and that it in addition participates in the regulation of K^+ ^homeostasis. Figure 6 provides a model of the ion transport mechanisms, which we suggest are present in the duct cell. In this model a Na^+^-K^+^-ATPase in the basolateral cell membrane pumps Na^+ ^out of the cell and thereby provides the driving force for apical uptake of Na^+ ^through channels. The epithelial sodium channel (ENaC) is a likely candidate mediating this apical Na^+ ^uptake [43-45]. Na^+ ^exits the cell through the pump. K^+ ^is secreted through apical channels, and is recycled for the pump across the basolateral cell membrane. It is highly probable that ROMK channels, known to mediate K^+ ^secretion in the mammalian collecting duct system [42], and shown to be expressed in amphibian pronephric ducts [18], mediate K^+ ^secretion across the apical cell membrane. The active transport of Na^+ ^would create a lumen-negative transepithelial potential, and Cl^- ^would presumably follow passively through the paracellular pathway of this epithelium [45]. This is to our knowledge the first characterization of the pronephric duct-the precursor of the collecting duct system- which provides detailed information on cell structure and the basic mechanisms for ion transport.

## Methods

### Animals

Specimens of the Mexican axolotl *Ambystoma mexicanum *(Shaw and Nodder, 1798) came from the animal stable of the August Krogh Building, part of the Campus Animal Research Facility at University of Copenhagen. Staging were performed according to [46] for embryos and the larvae were designated according to the degree of limb development as defined by the *Ambystoma *Genetic Stock Center, University of Kentucky; http://www.ambystoma.org. Larvae used for experiments, were in the stage 44 to 54. They were euthanized by decapitation, followed by brain destruction, and were subsequently either prepared for histology or pronephric ducts (o.d. 50-70 μm, dissected length 300-1000 μm) were free hand dissected at 6°C and prepared for microperfusion experiments or immunolabeling. The pronephric ducts were dissected from the region in front of the mesonephros. Dissections were performed in media containing (in mmol/l): 75 NaCl, 20 NaHCO_3_, 3.0 KCl, 1.8 CaCl_2_, 1.0 MgSO_4_, 0.8 Na_2_HPO_4_, 0.2 NaH_2_PO_4_, 5.5 glucose, 3.3 glycine, 0.4 PVP, 5.0 HEPES, titrated to pH 7.8 with NaOH.

### Histology, Light and Transmission Electron Microscopy

Dissections were performed for every developmental stage of post-hatched axolotls from stage 44 to 57. In addition, we examined pronephric development in embryos. Light microscopic imaging of live and dissected larvae was performed using Zeiss Stemi 2000-CS and Leica MZ 16 microscopes equipped with an Infinity X Digital Camera (DeltaPix, Denmark). For histology, a total of 31 larvae in the stage 44-54 and 19 embryos from stage 21 to 44 were used. Specimens were fixed for 12 hours at room temperature in an aldehyde fixative containing: 1.2% glutaraldehyde, 1% paraformaldehyde, 0.05 mol/l sucrose and 0.05 mol/l sodium cacodylate buffer (pH 7.4) and subsequently rinsed and stored in 0.05 mol/l sodium cacodylate buffer with 0.05 mol/l sucrose. Following 1 hour's post fixation in 2% OsO_4 _with 0.1 mol/l sodium cacodylate, specimens were dehydrated through a graded series of ethanol and propylenoxide and embedded in Araldite. For light microscopy, 1.5 μm sections were cut with glass knives on a Leica ultramicrotome EM UC6 and stained with toluidine blue. Ultrathin sections for transmission electron microscopy were cut on the same microtome with a Diatome diamond knife and subsequently stained with uranyl acetate and lead citrate. Transmission electron microscopic images were acquired using JEOL 100SX and JEOL JEM 1011 transmission electron microscopes. Kodak negatives obtained from the JEOL 100SX were digitized using an Epson Perfection 4990 Photo scanner. The JEOL JEM-1011 was equipped with a GATAN digital camera. Digital images were optimized for contrast and color using CorelDraw X4.

### Microperfusion and cellular impalements

Pronephric ducts were transferred to a bath chamber mounted on an inverted microscope and perfused *in vitro *with a set of pipettes made to fit the diameter of the tubules [37-39]. The tubule perfusion system used (Luigs & Neumann, Germany) was designed for accurate adjustment and movement of concentric pipettes [47]. Holding and perfusion pipettes were hand made from glass tubing (Drummond Scientific Company, PA, USA; holding pipettes: o.d. 2.1 mm, i.d. 1.6 mm; perfusion pipettes: o.d. 1.2 mm, i.d. 1.0 mm) in a microforge equipped with a microscope (SM II/1 Puller from Luigs & Neumann, Germany). The tubules were perfused with a single-barrelled perfusion pipette containing a small glass capillary (o.d. 0.3 mm, i.d. 0.2 mm, Drummond Scientific Company, PA, USA) connected to a manual Hamilton valve (Hamilton Co., NV, USA) with a four-way distribution system. Fluid exchange during luminal perfusion experiments was made through this capillary, the tip of the capillary being placed close to the opening of the perfusion pipette, ensuring fast fluid exchange in the tubule. Luminal perfusion was performed either by hand through a syringe connected to one of the ports in the valve or it was gravity-driven through a port connected to a fluid filled reservoir. The pressure applied was adjusted by monitoring tubule diameter, ensuring that the tubule neither collapsed nor over expanded. The bath was perfused at 8 ml/min and fluid exchange was performed through beakers attached to the outside of the Faraday cage, which surrounds the microperfusion set-up.

Perfusions of tubule bath and luminal fluid were carried out at room temperature with a perfusion solution containing (in mmol/l): 75 NaCl, 25 NaHCO_3_, 3.0 KCl, 1.8 CaCl_2_, 1.0 MgSO_4_, 0.8 Na_2_HPO_4_, 0.2 NaH_2_PO_4_. The perfusion solution was equilibrated with 1.8% CO_2 _in O_2 _and had a measured pH of 7.8. Experimental solutions with different sodium and potassium concentrations were prepared from this control solution. In the high K^+ ^solution, the K^+ ^concentration was raised to 20 mmol/l by equimolar substitution with Na^+^. In the low Na^+ ^solution with a [Na^+^] of 6.8 mmol/l, Na^+ ^was replaced by choline or N-methyl-D-glucamine (NMDG^+^) titrated with HCl.

Pronephric duct cells were impaled across the basal cell membrane with KCl (1-3 mol/l) filled glass microelectrodes (R_electrode _≈ 100 MΩ) and the membrane potential (V_m_) was recorded with respect to the grounded bath. The microelectrodes were pulled from borosilicate glass with filament (Clark Electromedical, UK) on a vertical electrode puller (Narishige, Japan). Impalements were achieved by placing the microelectrode tip against the basal surface of the cell and gently taping the micromanipulator (Leitz, Germany) on which the electrodes were mounted. Voltage recordings were made by a WPI Duo 773 electrometer (World Precision Instruments, USA) and digitized by a PowerLab/4S data acquisition system (ADInstruments, Australia). The recording of V_m _was accepted if the impalement was achieved by a sudden change in the potential read by the electrode and if the impalement was stable.

The results are based on 64 cell impalements made in 22 pronephric ducts dissected from 22 axolotl larvae in the stage 46-54. Figures were made in Origin 7.5 (Microcal, USA) and CorelDRAW X4.

### Immunolabeling, Confocal Laser Scanning Microscopy and 3D reconstruction

For identification and localization of the Na^+^-K^+^-ATPase, three separate immunolabeling experiments were conducted with equal results covering larvae in stages 46-54. In each experiment, pronephric ducts were isolated from four to five specimens and subsequently fixed on ice for approximately 60 minutes in 3% paraformaldehyde buffered to pH 7.4 with 0.1 mol/l sodium cacodylate. After being rinsed in PBS (perfusion solution), the tissue was incubated overnight at 4°C in PBS containing 10% goat serum (Invitrogen, CA, USA), 1% triton-X and the Na^+^-K^+ ^ATPase monoclonal mouse antibody α5-IgG (10 μg/ml). The α5 antibody developed by D.M. Fambrough was obtained from the Developmental Studies Hybridoma Bank developed under the auspices of the NICHD and maintained by The University of Iowa, Department of Biology (Iowa City, IA 52242). Following an extensive wash in PBS, the pronephric ducts were incubated with Alexa-488-conjugated goat anti-mouse IgG (1:100, Invitrogen, CA, USA) overnight at 4°C. Following rinses in PBS, the tissue was mounted on glass coverslips in Vecta shield (Vector Laboratories Inc., CA, USA). In some preparations, the renal tubules were incubated in DAPI (1:250, Invitrogen, CA, USA) for approximately 5 min and washed in PBS, prior to mounting. Image acquisition was performed on a Leica DM RXE 6 TL inverted microscope equipped with a Leica TCS SP2 AOBS confocal laser scanning unit, using the 488 nm line of an argon/crypton laser. The image series were processed and edited in the 3-D reconstruction software IMARIS (Bitplane AG, Zürich, Switzerland). The confocal images are based on 150-170 optical sections of a Z-series performed at intervals of 0.3-0.5 μm. All control preparations were negative for immunostaining.

## Abbreviations

bl: basal labyrinth; ci: ciliated tubule; co: coelom; dt: distal tubule; gl: glomus; is: ciliated intermediate segment; nc: notochord; ne: nephrostome; nu: nucleus; mr: intercalated mitochondria-rich cell; mu: muscle; mv: microvilli; pd: pronephric duct; pt: proximal tubule; V_m_: membrane potential; wd: Wolffian duct.

## Authors' contributions

NM conceived and designed the study. BMH and NM fixed larvae and performed the dissections for the structural investigation. BMH sectioned specimens, and made LM and TEM investigations with help from ÅJ and NM. NM made the microperfusion experiments and microelectrode impalements. KAH, LRP and NM carried out immunostaining experiments and KAH performed CLSM and prepared the 3D images. All authors participated in discussions and interpretation of the data. NM wrote the paper with inputs from the other authors. All authors read and approved the final version of the manuscript.
